# Depletion of Uric Acid by Continuous Kidney Replacement Therapy

**DOI:** 10.1159/bpu/abpag008

**Published:** 2026-09-01

**Authors:** Seolhyun Lee, William Vasquez Espinosa, Tammy L. Sirich, Timothy W. Meyer

**Affiliations:** The Departments of Medicine, Veterans Affairs Palo Alto Healthcare System and Stanford University, Palo Alto, CA, USA

**Keywords:** Uric acid, Continuous kidney replacement therapy

## Abstract

**Introduction:**

Uric acid levels are commonly elevated in patients maintained on hemodialysis because hemodialysis provides a lower time-averaged uric acid clearance than the native kidney. However, continuous kidney replacement therapy (CKRT) provides higher time-averaged clearances of small solutes than hemodialysis. We therefore examined whether the high small solute clearance provided by CKRT would reduce plasma uric acid levels below normal.

**Methods:**

We measured plasma levels of uric acid and other selected solutes in 10 patients initiating CKRT and in 17 patients maintained on CKRT for 5–110 days. We estimated solute generation rates from their removal rates. The removal rates of uric acid and phosphate were estimated from their plasma levels, while those of urea and creatinine were calculated directly from their effluent levels. CKRT prescription met current guidelines (24 ± 2 mL/kg/h in patients initiating CKRT and 26 ± 3 mL/kg/h in patients maintained longer on CKRT).

**Results:**

Plasma uric acid levels declined from 8.7 ± 3.0 mg/dL to 1.8 ± 0.5 mg/dL after 2–4 days of initiating CKRT (*p* < 0.001) and were similarly low at 1.7 ± 0.6 mg/dL during ongoing CKRT. Plasma uric acid levels at both intervals were below the normal range. The average uric acid removal rate was not different at 2–4 days post-initiation (922 ± 367 mg/day) and during ongoing CKRT (855 ± 346 mg/day).

**Conclusion:**

CKRT prescribed according to current guidelines reduces uric acid levels below normal. The clinical consequences of low uric acid levels in this setting are unknown.

## Introduction

Continuous kidney replacement therapy (CKRT) has become the standard treatment for critically ill patients who are hemodynamically unstable [1–4]. Like hemodialysis, CKRT removes small solutes non-selectively. In comparison with intermittent hemodialysis, CKRT provides higher time-averaged clearances of these solutes. The higher clearances achieved by CKRT may cause depletion of valuable small solutes which are normally reabsorbed by the kidney tubules following glomerular filtration. Previous studies have evaluated the removal of valuable solutes including phosphate, amino acids, carnitine, vitamins, and trace elements by CKRT, and some of these solutes are commonly replaced [5–11]. Other studies have shown that uric acid is also effectively cleared by CKRT, and a recent study suggested that the fractional reduction in uric acid levels following CKRT initiation could serve as an indicator of adequate treatment [12–16]. The current study further evaluated the effect of CKRT on plasma levels of uric acid. Intermittent hemodialysis provides a lower effective clearance of uric acid than the native kidney. As a result, plasma uric acid levels are commonly above normal in hemodialysis patients [17, 18]. The current study assessed whether the higher clearance of small solutes provided by CKRT would in contrast reduce plasma uric acid levels below normal.

## Methods

### Human Subjects

The study was observational and prospective and conducted at a single center. Subjects were recruited from Stanford Health Care between April 2024 and April 2025. Two groups of patients were enrolled. The first group consisted of patients initiating CKRT from whom blood samples were collected prior to initiating treatment and again during the second to the fourth day of treatment. The second group comprised patients who had been maintained on CKRT for at least 5 days and from whom a single blood sample was collected at the time of study enrollment. Samples of effluent were collected at the same time as the blood sample after 2–4 days of treatment in the patients initiating CKRT and as the single blood sample in the patients receiving ongoing CKRT. Patients were eligible for enrollment if they were 18 years of age or older and had acute kidney injury requiring CKRT. Patients were excluded if they had end-stage kidney disease, were admitted to the hospital already on CKRT, were participating in a clinical trial, were moving to comfort-oriented care, or had conditions expected to markedly alter uric acid metabolism, including tumor lysis syndrome, Amanita toxicity, or use of uric acid lowering therapies (online suppl. Fig. 1; for all online suppl. material, see https://doi.org/10.1159/bpu/abpag008).

### Laboratory Methods and Statistics

Plasma uric acid levels were measured using a spectrophotometric method by the clinical laboratory. The laboratory’s normal ranges for adult females and males are 2.4–5.7 mg/dL and 3.4–7.0 mg/dL, respectively. Plasma and effluent levels of urea nitrogen (ureaN), creatinine, and phosphate were also measured by the clinical laboratory. The removal rates for ureaN and creatinine by CKRT were calculated as the product of the daily effluent volume and the solute concentration in an effluent sample. The removal rates of uric acid and phosphate were estimated as the product of their plasma concentrations multiplied by the daily effluent volume as the clinical laboratory could not measure uric acid or phosphate levels in effluent fluid.

### Statistical Analysis

Solute levels before and after 2–4 days of CKRT were compared using the paired *t* test. Solute levels and estimated solute removal rates after 2–4 days of CKRT and during longer ongoing CKRT were compared using the unpaired *t* test.

## Results

The characteristics of the study subjects are summarized in Table 1. The CKRT prescription in all cases was continuous venovenous hemodiafiltration and provided an effluent flow of at least 20–25 mL/kg/h as recommended by the Kidney Disease Improving Global Outcomes (KDIGO) Guidelines [19]. The total effluent was comprised of approximately half dialysate and half ultrafiltrate with the replacement fluid typically divided evenly between pre- and post-filter.

**Table 1.** Characteristics of the participants

​	Initiating CKRT (*n* = 10)	Ongoing CKRT (*n* = 17)
Female/male ratio	2/8	5/12
Weight, kg	88±19	81±28
Duration CKRT, days	–	22±27
CKRT effluent, mL/kg/h	24±2	26±3
Protein intake, g/day	–	108±44

Values are mean ± SD. The CKRT effluent rate is the value recorded on the day of 2nd sample collection for the patients initiating CKRT. Protein intake during CKRT was obtained from Clinical Nutrition Service notes detailing the protein equivalents received by enteral or parenteral nutrition over the past 3–7 days. This information was not reliably available on the day of CKRT initiation.

Table 2 shows the effects of initiating CKRT on plasma solute levels. Initiating CKRT rapidly reduced plasma levels of uric acid as well as urea nitrogen (ureaN) and creatinine. Within 2–4 days of CKRT initiation, plasma uric acid decreased by an average of 78 ± 8%, falling below the lower limit of the laboratory’s reference range in every case. Plasma ureaN, creatinine and phosphate levels also decreased rapidly following the initiation of CKRT. Table 2 further shows values obtained in patients receiving ongoing CKRT. Uric acid levels remained similar to those observed after 2–4 days, again being below the reference range in every case. Creatinine levels were lower in patients maintained on CKRT for 5 or more days than in patients who had received CKRT for 2–4 days. In contrast, ureaN and phosphate levels were not significantly different.

**Table 2.** Plasma solute levels

​	Plasma solute levels			*p* value	
	initiating CKRT (*n* = 10)		ongoing CKRT (*n* = 17)	pre vs. 2–4 days	2–4 days vs. ongoing
	pre	2–4 days			
Uric acid, mg/dL	8.7±3.0	1.8±0.5	1.7±0.6	0.000	0.780
UreaN, mg/dL	58±26	30±12	32±12	0.001	0.679
Creatinine, mg/dL	4.3±2.4	2.3±0.8	1.2±0.4	0.012	0.001
PO_4,_ mg/dL	5.1±1.6	2.9±0.6	3.4±0.9	0.002	0.089

Values are mean ± SD.

Calculated solute removal rates in patients receiving CKRT are summarized in Table 3. Uric acid removal rates observed during ongoing CKRT were similar to those observed soon after initiation of (855 ± 346 vs. 922 ± 367 mg/day, respectively), as were the removal rates of ureaN and phosphate. In contrast, the creatinine removal rate was notably lower in the group receiving ongoing CKRT.

**Table 3.** Estimated solute removal rates

​	Initiating CKRT (*n* = 10)	Ongoing CKRT (*n* = 17)	*p* value
Uric acid, mg/day	922±367	855±346	0.646
UreaN, mg/day	14,461±6,096	15,169±6,164	0.775
Creatinine, mg/day	1,075±443	605±329	0.011
Phosphate, mg/day	1,437±356	1,651±581	0.247

Values are mean ± SD.

## Discussion

The kidney reabsorbs many valuable solutes from the glomerular filtrate so that very little is lost in the urine. In contrast, kidney replacement therapies remove small solutes non-selectively. Intermittent hemodialysis provides high clearances of small solutes for only a fraction of each week. As a result, loss of valuable small solutes is usually not clinically significant, and laboratory testing or replacement of such solutes is not routinely required in hemodialysis patients [7].

CKRT prescribed according to current guidelines provides a higher time-averaged clearance of small solutes than intermittent hemodialysis [20–22]. However valuable small solutes may also be removed to a greater extent by CKRT than hemodialysis. Potential depletion of such solutes, including amino acids, vitamins, carnitine, and trace elements has been evaluated [6–9]. Guidelines include recommendations for replacing some of them [23–25].

We found that uric acid may also be depleted by CKRT. Uric acid is an end product of purine metabolism in humans and other hominids [26] Uric acid is not protein-bound and is filtered by the glomerulus at a rate equal to the GFR. However the majority of the filtered uric acid is reabsorbed in the proximal tubule so that the urinary uric acid clearance in healthy humans is approximately 10 mL/min/1.73 m^2^ [27–29]. Conventional thrice weekly hemodialysis provides a lower effective time-average uric acid clearance due to uric acid’s limited dialytic clearance and volume of distribution. Uric acid levels in patients maintained on intermittent hemodialysis are therefore above normal and would be even higher if the proportion of uric acid removed by the intestine did not increase [18]. However CKRT removes uric acid much more effectively than intermittent hemodialysis. The uric acid clearance by CKRT is close to the effluent flow rate [12–14]. The CKRT received by patients in our study thus provided average uric acid clearances more than 25 mL/min/1.73 m^2^. As a result, uric acid levels were reduced considerably below normal after 2–4 days on CKRT and remained low in patients continuing on CKRT. Of note, a single patient receiving a CKRT dose of approximately 90 mL/kg/h to treat hyperammonemia was excluded from our analysis. The uric acid level in this patient declined from 8.8 mg/dL to 0.4 mg/dL after 90 h on CKRT. This profound depletion suggests the need for further evaluation of the effects of removal of uric acid and other metabolites in patients receiving high-dose CKRT.

The difference in the effects of CKRT and hemodialysis on uric acid is similar to the difference in the effects of these modalities on phosphate. Phosphate like uric acid is partially reabsorbed after glomerular filtration in the native kidney. A difference between phosphate and uric acid is that phosphate reabsorption is closely regulated, so that phosphate levels are maintained normal by a reduction in the kidney phosphate clearance when intake is low and an increase in the kidney phosphate clearance when intake is high. Intake is high enough in most people so that dialysis does not remove enough phosphate to maintain normal levels, and phosphate binders are usually prescribed to reduce gastrointestinal phosphate absorption. In contrast, the high clearance provided by CKRT removes more phosphate than is provided by standard hospital nutrition, and phosphate is commonly administered by intermittent intravenous dosing or added to replacement fluids. Because phosphate supplementation was not standardized, we could not assess its effect on plasma phosphate levels in our study. We are not aware of an organic solute other than uric acid for which hemodialysis provides a lower effective clearance than the native kidney while CKRT provides higher clearance and thus reduces the solute’s level below normal.

Our estimates of solute removal rates allow us to make inferences about solute generation rates. Our estimated uric acid removal by CKRT was higher than the uric acid generation rate reported in healthy humans [30–32]. Based on uric acid having a clearance close to the CKRT effluent rate and a reported volume of distribution between the extracellular fluid and total body water volumes, we presume that plasma uric acid levels came close to equilibrium within 2 days after initiation of CKRT, as depicted in online supplementary Figure 2. Thus, the high removal rate estimated at 2–4 days did not simply reflect removal of uric acid from an increased body pool. In subjects with normal kidney function, about one third of the uric acid generated is cleared by the intestine. We suppose that gastrointestinal removal of uric acid is if anything lower than normal in ICU patients with low plasma uric acid levels. Under these assumptions, the estimated removal rates raise the possibility that uric acid generation may be increased in these patients. As standard parenteral or enteral nutrition lacks purines, we hypothesize that increased generation rate results from increased endogenous purine degradation [33, 34]. This hypothesis however must be advanced with caution. The removal rates for uric acid and phosphate presented in Table 3 were based on their plasma concentrations, whereas those of ureaN and creatinine were calculated directly from their effluent concentrations. We did find that effluent to plasma ratios for urea and creatinine in the current study were close to 1.0 (online suppl. Table 1). This, together with prior reports of uric acid clearance by CKRT, suggests that calculations based on its plasma concentration did not greatly overestimate the rate of uric acid removal [12–15]. Our estimation of uric acid removal was based on its plasma concentration measured at a single time point and on the assumption of a stable volume of distribution. We also assumed that urinary uric acid removal was negligible in patients who still produced urine.

Creatinine has a larger volume of distribution than uric acid and its levels may take longer to approach equilibrium. Consequently, removal from an increased body pool may have contributed to the creatinine removal rate at 2–4 days. The very low creatinine removal rate observed with ongoing CKRT presumably resulted from low creatinine production as has previously been reported in critically ill patients [35, 36]. As with creatinine, reduction of an initially high body pool may have contributed to the ureaN removal rate observed shortly after initiation of CKRT. The ureaN removal rate of 14.7 g/day with ongoing CKRT is consistent with the recorded provision of nutrition including an average of 108 g/day of protein and/or amino acids.

We do not know whether the uric acid depletion we observed is harmful. Plasma uric acid levels are less than 1 mg/dL in most mammals, whereas uric acid levels in humans and other hominids are 3–10 times higher. High uric acid levels in hominids are the combined result of the loss of uricase activity and reabsorption of uric acid in the proximal tubule [27]. The evolutionary value of higher uric acid levels in hominids is debated. One theory suggests it allowed more rapid storage of energy from fruits in hominid ancestors [37]. It has also been suggested that uric acid serves as an antioxidant and has particular value in limiting oxidant-mediated neurologic injury [27, 38, 39]. People with hypouricemia resulting from genetic defects in proximal tubular uric acid reabsorption develop exercise-related acute kidney injury and may have an increased risk of developing CKD [40, 41]. Lack of uric acid reabsorption could potentially cause injury in pig-to-human kidney transplants [42]. However we did not find reports that genetic hypouricemia increases mortality.

Our study has important limitations. First, we did not measure uric acid production and so could not compare production to removal rates. Second, we did not directly measure uric acid concentration in the effluent and estimated uric acid removal rates from plasma uric acid concentrations. Third, our study was too small to relate uric acid depletion to any clinical outcomes.

Prescription of an effluent rate of 25–30 mL/kg/h to achieve a delivered rate of 20–25 mL/kg/h is based on trials showing that higher doses provided no benefit [19, 20, 43, 44]. Proposed trials may address the question of whether a lower small solute clearance by CKRT could be equally effective [45, 46]. Further research might alternatively examine possible loss of a wider range of potentially valuable solutes with the modern standard practice of this very effective therapy.

In conclusion, we found that plasma uric acid levels are markedly reduced in patients maintained on CKRT. It is not clear that low uric acid levels are harmful in this setting and we are thus not at present motivated to restore them toward normal.

## Acknowledgments

The authors wish to acknowledge the help of the Stanford Dialysis and ICU Nursing Staff in enrolling patients in this study.

## Statement of Ethics

This study protocol was reviewed and approved by Stanford University Internal Review Board, Approval No. 33656. Written informed consent was obtained from participants.

## Conflict of Interest Statement

The authors report no conflicts of interest.

## Funding Sources

The study was supported by NKF Young Investigator Award to Dr. S.L. and by National Institutes of Health awards (R01 DK101674 to T.W.M. and R01 DK118426 and R01 NS054791 to T.L.S.). The funding agency had no role in the design, execution, analysis, or interpretation of the data, or in the decision to submit the manuscript for publication.

## Author Contributions

S.L.: conceptualization, data curation, formal analysis, investigation, methodology, project administration, writing – original draft, and writing – review and editing. W.V.E.: data curation, investigation, and methodology. T.L.S.: conceptualization, data curation, formal analysis, funding acquisition, methodology, project administration, resources, writing – original draft, and writing – review and editing. T.W.M.: conceptualization, data curation, formal analysis, funding acquisition, investigation, methodology, project administration, supervision, validation, writing – original draft, and writing – review and editing.

## Data Availability

The data that support the findings of this study are not publicly available due to confidentiality reasons but are available from the corresponding author upon request.
